# Supplementary material for: A data-adaptive method for investigating effect heterogeneity with high-dimensional covariates in Mendelian randomization
Source: BMC Med Res Methodol. 2024 Feb 10;24:34. doi: 10.1186/s12874-024-02153-1 (PMC10858611; doi:10.1186/s12874-024-02153-1)
Supplement: Supplementary file 1 — Additional file 1. [file 12874_2024_2153_MOESM1_ESM.pdf]

A data-adaptive method for investigating effect heterogeneity with high-dimensional covariates in Mendelian randomization Supplementary Materials

Haodong Tian[1]*, Brian D. M. Tom[1], Stephen Burgess[1,2]†

[1] MRC Biostatistics Unit, School of Clinical Medicine,
University of Cambridge, Cambridge, UK

[2] British Heart Foundation Cardiovascular Epidemiology Unit,
Department of Public Health and Primary Care,
University of Cambridge, Cambridge, UK

Supplementary Table

Abbreviation	Meaning
hip	Hip circumference
wt	Weight
dbp	Diastolic blood pressure
waist	Waist circumference
monos	Monocyte count
leuc	Leucocyte count
vitcap	Vital capacity
bmi.1	Body mass index
pef	Peak expiratory flow
whtr	Waist to height ratio
sbp	Systolic blood pressure
neutros	Neutrocyte count
urianac	Urinary sodium
whr	Waist to hip ratio
uriamac	Urinary microalbumin
handgpr	Hand grip strength – right
eosins	Eosinophil count
hemcrit	Haematocrit
lymphs	Lymphocyte count
uriakc	Urinary potassium
pulse	Pulse rate
ages	Age at survey
handgpl	Hand grip strength – left
uriacc	Urinary creatinine
ht	Height
platelet	Platelet count
rbc	Red blood cell count
hemglob	Haemoglobin

Supplementary Table S1: Abbreviations of the variables considered in the UK Biobank application example. As body mass index is the exposure, stratifying on body mass index is equivalent to a non-linear Mendelian randomization analysis.

*haodong.tian@mrc-bsu.cam.ac.uk
†sb452@medschl.cam.ac.uk

Supplementary Figure

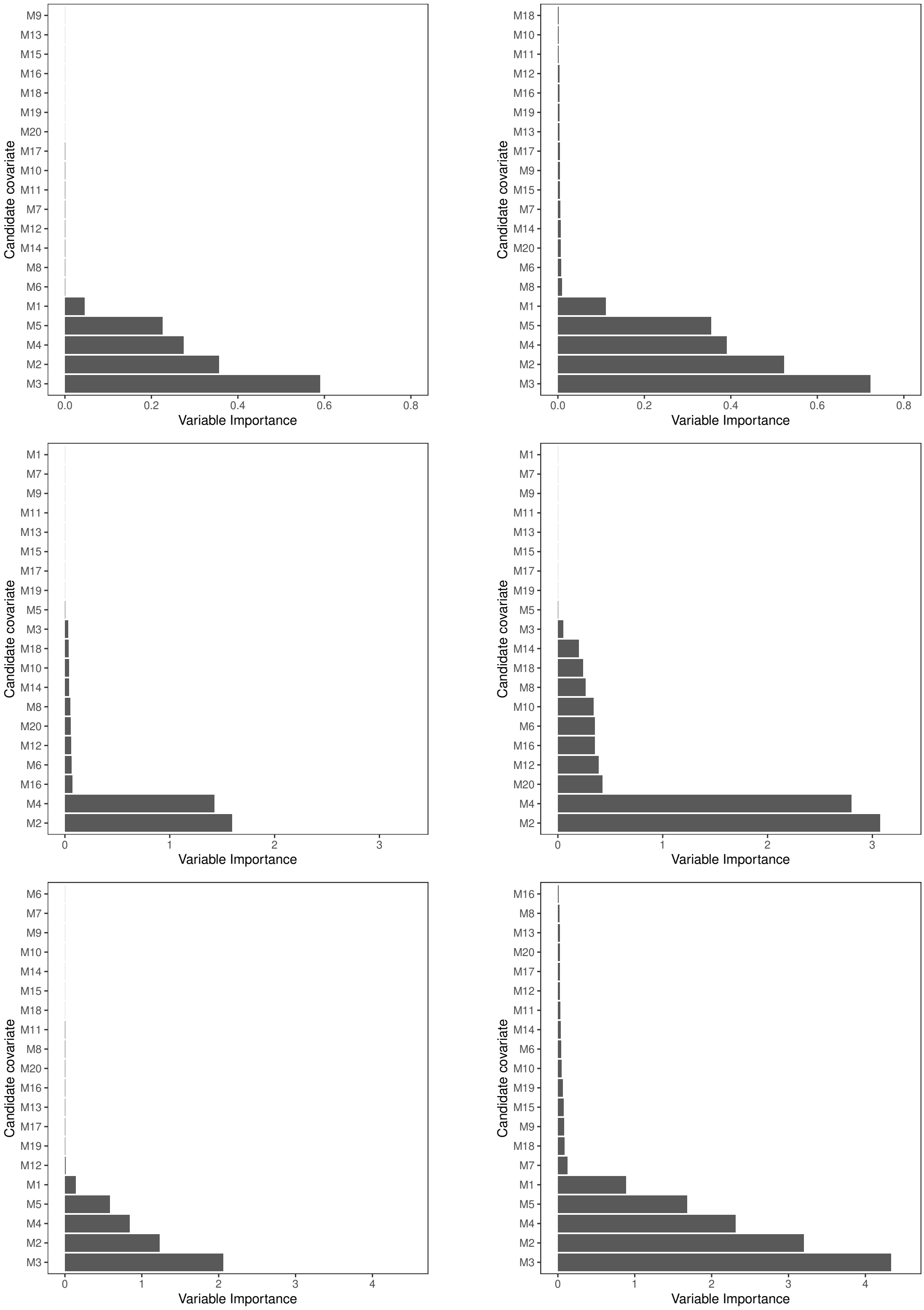

Supplementary Figure S1: Variable importance (VI) measurements for the doubly-ranked method with random forest. Left panels: VI measurements calculated using individual effect labels (that is, the true individual effects), based on changes in mean squared error. Right panel: VI measurements calculated without individual effect labels, based on changes in estimates. The top, middle, and bottom results correspond to a single randomly chosen simulated dataset under scenario A, B, and C with the strength of modification 0.5, respectively. The true effect modifiers are M1-M5.

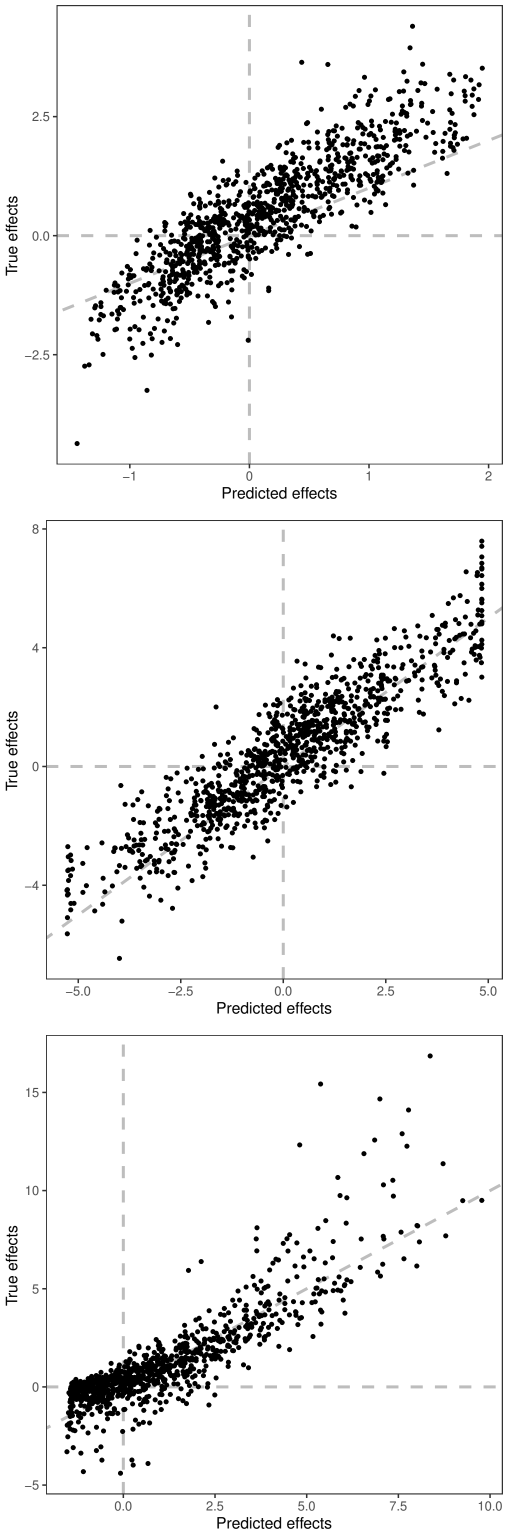

Supplementary Figure S2: Scatterplot displaying the predicted effects and true effect values for 1000 randomly selected individuals from the testing set for the doubly-ranked method with random forest. The top, middle, and bottom plots correspond to a single randomly chosen simulated dataset under the simulation scenarios A, B, and C with a strength of modification of 0.5, respectively.

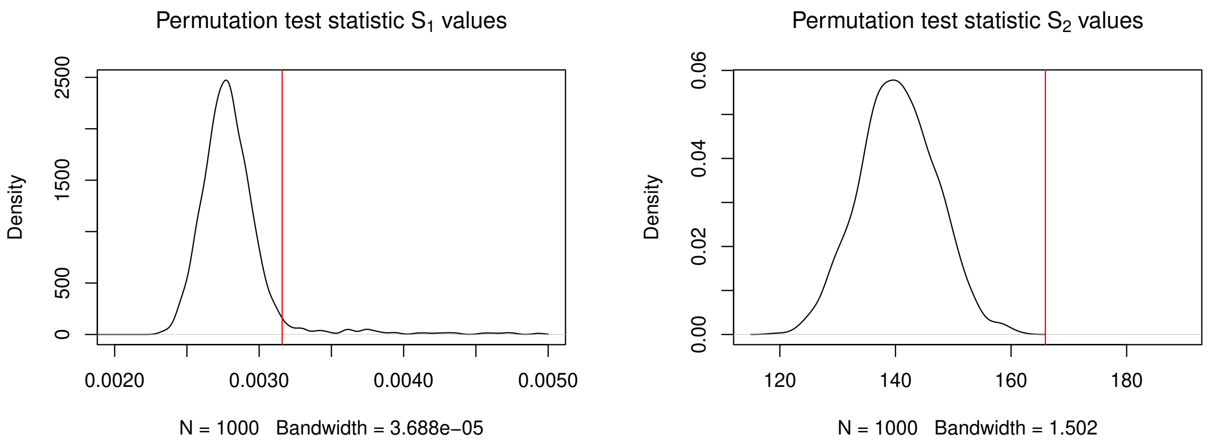

Supplementary Figure S3: The kernel smoothed density of the under-null samples of the permutation test statistics S_1 (left) and S_2 (right) with 1000 permutations. The statistic values for the unpermuted data are shown by the red lines. The bandwidth is decided by Silverman's rule of thumb.

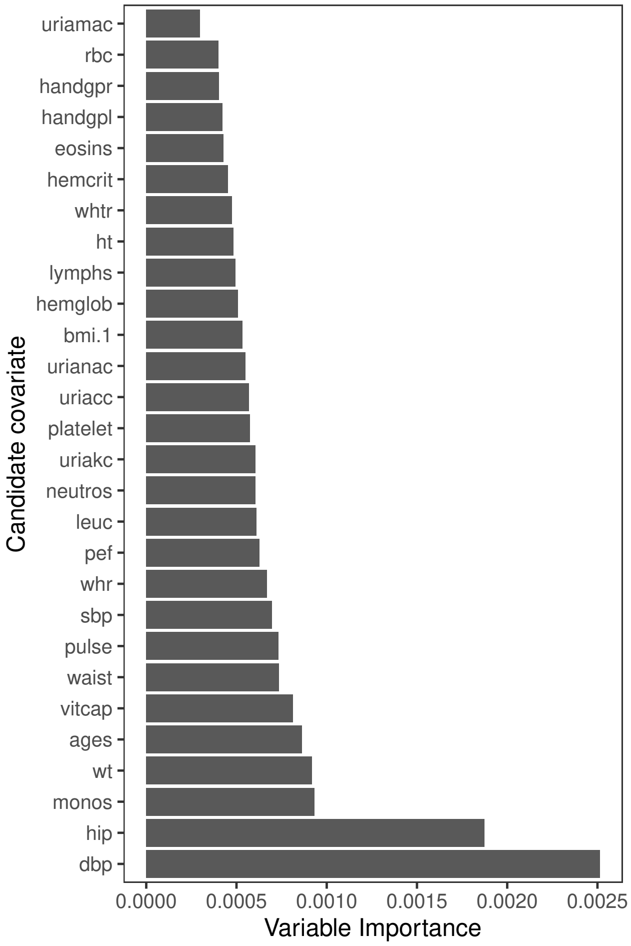

Supplementary Figure S4: Variable importance measures in the UK Biobank example for 28 candidate covariates.

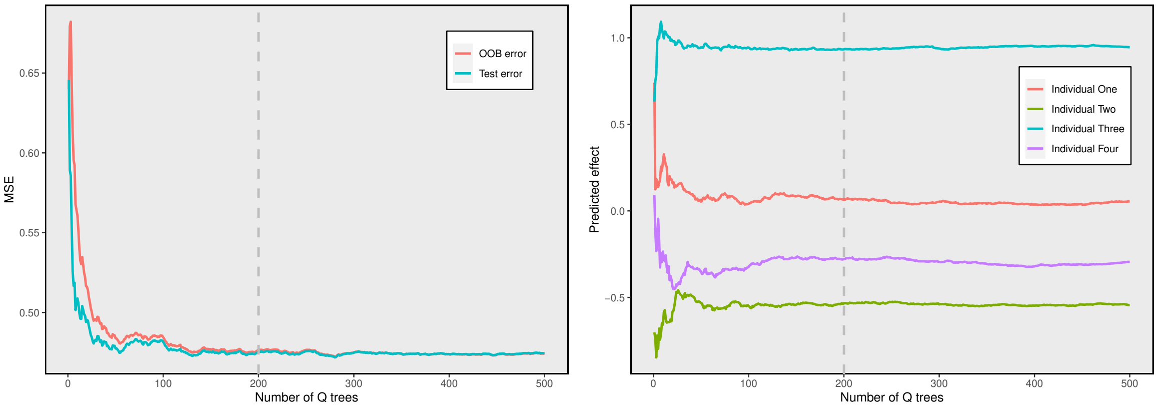

Supplementary Figure S5: Left panel: the mean squared error (MSE) of the OOB samples (red curve) and testing subset samples (blue curve) with increasing numbers of Q trees for the simulation study. Right panel: the predicted values of four randomly selected samples of the testing subset with increasing numbers of Q trees for the simulation study. OOB: out-of-bag.

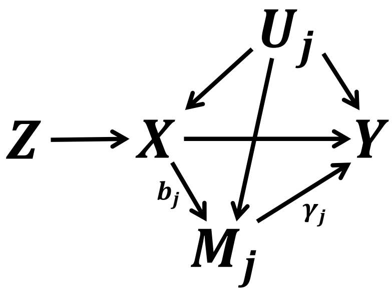

Supplementary Figure S6: Directed acyclic graph (DAG) demonstrating the variable relationships in simulation. Z, X, Y, U_j, M_j represents the instrument, the exposure, the outcome, the j-th confounders and the j-th covariate (here a mediator). b_j represents the effect of the exposure on the j-th covariate, and γ_j represents the modification effect of the j-th covariate on the direct effect of the exposure on the outcome.

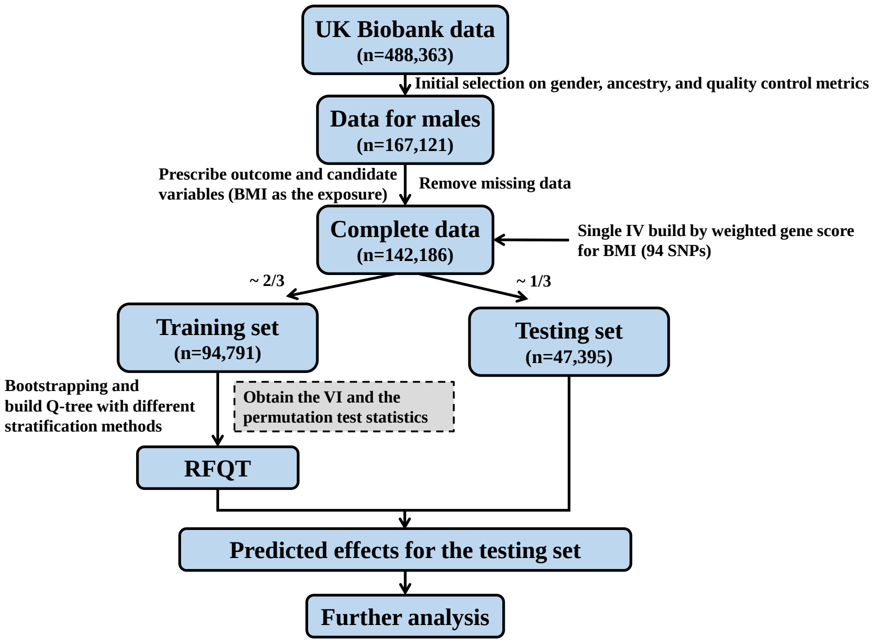

Supplementary Figure S7: Diagram demonstrating analysis flow for the UK Biobank data. IV: instrumental variable. RFQT: random forest of Q trees. VI: variable importance.

Supplementary Text

We have included a supplementary simulation to encompass more comprehensive scenarios involving the covariates outlined in Table 1. While the main text focused on covariates belonging to scenarios 1, 5, 7, and 9 of Table 1, this supplementary simulation considers each covariate belonging to one of the remaining scenarios (scenarios 2, 3, 6, and 8 of Table 1). Note that scenario 4 of Table 1 corresponds to an ill-defined Directed Acyclic Graph (DAG) case and, therefore, will not be considered.

We consider the following data-generating model, where the individual index has been omitted for notational brevity,

$$X = 0.5Z + 0.5\sum_{j=1}^{10} M_j + 0.5\sum_{j=1}^{20} U_j + \epsilon_X \tag{S1}$$

$$M_j = \begin{cases} U_j + \epsilon_{M_j} & \text{for } j = 1, 2, \ldots, 10 \\ U_j + 0.5X + 0.5Y + \epsilon_{M_j} & \text{for } j = 11, \ldots, 15 \\ U_j + 0.5Y + \epsilon_{M_j} & \text{for } j = 16, \ldots, 20 \end{cases} \tag{S2}$$

$$Y = \left(0.5 + \sum_{j=1}^{5} \gamma_j M_j\right) X + 0.5\sum_{j=1}^{20} U_j + \epsilon_Y \tag{S3}$$

where Z, X, $\{U_j\}$, $\{M_j\}$ and Y are the instrument, the exposure, unmeasured confounders, the candidate covariates, and the outcome, respectively. $Z \sim \mathcal{N}(0, 1^2)$, $U_j \overset{\text{i.i.d}}{\sim} \mathcal{N}(0, 1^2)$; $\epsilon_X, \epsilon_Y, \epsilon_{M_j} \sim \mathcal{N}(0, 1^2)$; $\{\gamma_j\}$ are the modifier effects by each candidate covariate and $\gamma_j \overset{\text{i.i.d}}{\sim} \mathcal{N}(\gamma, 0.1^2)$ for $j = 1, \ldots, 5$, and $\gamma_j = 0$ otherwise. γ is the strength of modification. According to the structural model, each covariate will have a different definition expressed by a DAG. For $j = 1, \ldots, 5$, M_j is expressed by the DAG below (corresponding to scenario 3 of Table 1), where M_j can be interpreted as both a confounder and an effect modifier.

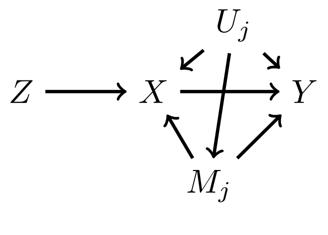

For $j = 6, \ldots, 10$, M_j is expressed by the DAG below (corresponding to scenario 6 of Table 1).

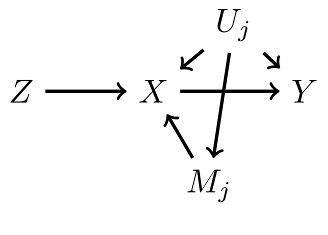

Similarly, we have the DAG below for $j = 11, \ldots, 15$ (corresponding to scenario 2 of Table 1), where M_j is a collider.

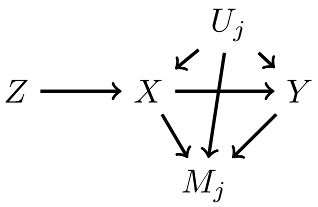

For $j = 6, \ldots, 10$, M_j is expressed by the DAG below (corresponding to scenario 8 of Table 1), where M_j is a downstream variable of Y, and therefore a collider.

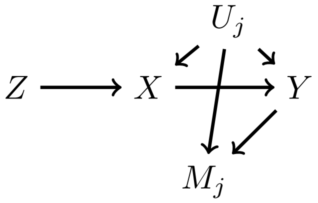

We applied our RFQT method to fit the simulation data independently 100 times. Each simulation generated 150,000 individual samples, with two-thirds used as the training set and the remaining one-third as the testing set. We compared the RFQT method using the doubly-ranked stratification with a no-stratification approach (i.e., estimating the average treatment effect). Considering six levels of modification strength ($\gamma = 0, 0.1, 0.2, 0.3, 0.4, 0.5$), we recorded the MSE regarding the direct effect (also the total effect in this supplementary simulation, as M_j is not a mediator) of the exposure on the outcome for the testing set in each independent simulation and for each modification strength case. The results are presented in Figure S8, revealing that our RFQT with the doubly-ranked stratification outperforms the no-stratification approach in terms of MSE across all modification strength cases. This conclusion, combined with findings from the main text, supports the claim that our RFQT is compatible with most covariate cases presented in Table 1.

We also take one simulation with the modification strength $\gamma = 0.5$ as an example to present the two variable importance measurements using RFQT with the doubly-ranked stratification. The results are provided in Figure S9. Our method can correctly identify the effect modifiers $M1$-$M5$ with the highest variable importance measurements among all covariate candidates.

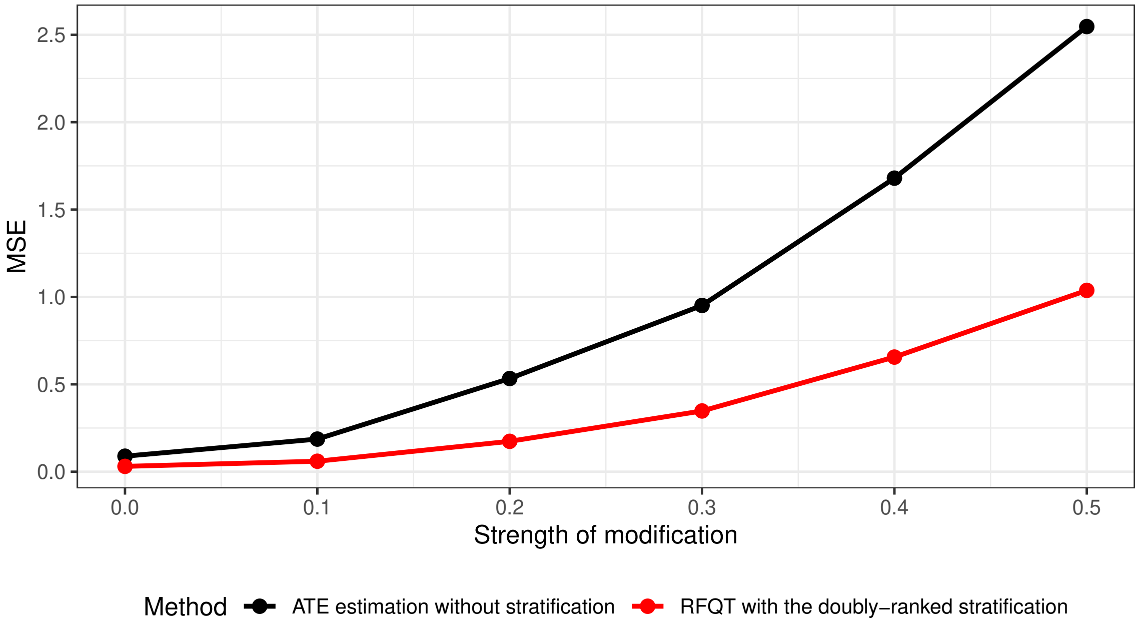

Supplementary Figure S8: Results of the supplementary simulation study showing mean squared error (MSE) of estimates with weak modification (strength of modification = 0.0) up to strong modification (strength of modification = 0.5). In each modification strength scenario, data are independently simulated 100 times, and the MSE represents the median value across simulations.

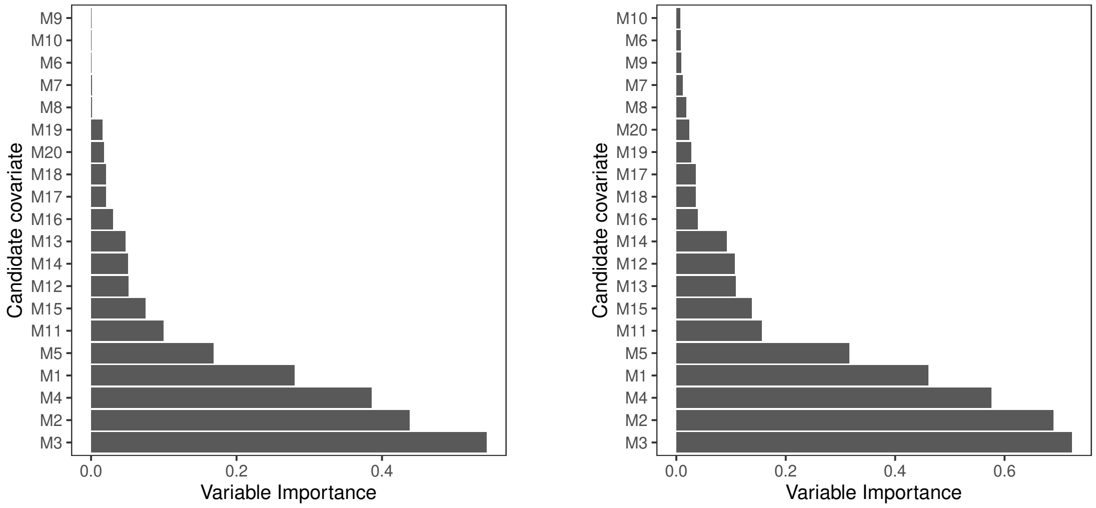

Supplementary Figure S9: Variable importance (VI) measurements for the doubly-ranked method with random forest. Left panels: VI measurements calculated using individual effect labels (that is, the true individual effects), based on changes in mean squared error. Right panel: VI measurements calculated without individual effect labels, based on changes in estimates. The true effect modifiers are M1-M5.
